# Efficacy and Risks of Posterior Vertebral Column Resection in the Treatment of Severe Pediatric Spinal Deformities: A Case Series

**DOI:** 10.3390/jcm14020374

**Published:** 2025-01-09

**Authors:** Emanuela Asunis, Chiara Cini, Konstantinos Martikos, Francesco Vommaro, Gisberto Evangelisti, Cristiana Griffoni, Alessandro Gasbarrini

**Affiliations:** 1 Department of Spine Surgery, IRCCS Istituto Ortopedico Rizzoli, 40136 Bologna, Italy; asunis.emanuela@gmail.com (E.A.); konstantinos.martikos@ior.it (K.M.); francesco.vommaro@ior.it (F.V.); gisberto.evangelisti@ior.it (G.E.); cristiana.griffoni@ior.it (C.G.); alessandro.gasbarrini@ior.it (A.G.); 2 Department of Biomedical and Neuromotor Sciences, Alma Mater Studiorum University of Bologna, 40126 Bologna, Italy

**Keywords:** adolescent idiopathic scoliosis, posterior vertebral column resection, Kyphoscoliosis, posterior arthrodesis

## Abstract

**Background/Objectives**: Surgery for adolescent idiopathic deformities is often aimed at improving aesthetic appearance, striving for the best possible correction. However, severe and rigid scoliotic curves not only present aesthetic issues but can also compromise cardiopulmonary health and cause early neurological impairment due to spinal cord compression, posing significant risks of morbidity and mortality if untreated. Conservative treatments are ineffective for severe curves, defined by scoliotic angles over 70° and flexibility below 30% on lateral bending X-rays. Treatment often requires invasive interventions, such as osteotomies and vertebral resections. In particular, posterior vertebral column resection (PVCR) has shown effectiveness in realigning vertebral structures in complex cases. This study describes the efficacy and risks of PVCR through a series of cases treated at our institution. **Methods**: This case series was conducted at the Rizzoli Orthopedic Institute in Bologna, involving eight pediatric patients with severe, rigid spinal deformities, operated upon between 2018 and 2023. The underlying pathologies included idiopathic kyphoscoliosis, neurofibromatosis type 1, Pott’s disease, and other congenital anomalies. Preoperative assessment included standard radiographs, magnetic resonance imaging, and computed tomography. During PVCR, motor and sensory evoked potentials were monitored to minimize neurological injury risk. Postoperative management included blood transfusions, antibiotic support, and early physiotherapy. **Results**: PVCR resulted in an average reduction in the Cobb angle from 86.3° preoperatively to 22.4° postoperatively, with a mean correction of 64%. The mean duration of the procedures was 337.4 min. Three patients had an uneventful postoperative course, while five developed complications, including infections and temporary neurological deficits, which were successfully managed. One patient developed an epidural hemorrhage that required emergency surgery for hematoma evacuation, with partial recovery. This study demonstrates the potential of PVCR for correcting rigid spinal deformities, highlighting the importance of postoperative management to minimize the associated risks. **Conclusions**: Posterior vertebral resection techniques offer significant promise in the correction of pediatric spinal deformities. Although ours is a small case series, it can provide important data for such treatment. Long-term monitoring is needed to fully understand the impact of these procedures and to further refine surgical techniques.

## 1. Introduction

Idiopathic adolescent deformities surgery is mainly aimed at improving aesthetic appearance, striving for the best possible correction. On the other hand, severe and rigid scoliotic curves not only present aesthetic problems but can also compromise cardiopulmonary health and cause early neurological impairment due to cord compression, with significant risks of morbidity and mortality if not treated properly. Another key aspect to consider is that these curves tend to progress over time and, in severe cases, can cause early neurological deficits [1]. For this reason, conservative treatment is not effective in the management of severe rigid deformities [1]. There is no unambiguous definition of a severe curve in the literature. However, a curve with a scoliotic angle of at least 70° has been described as ‘wide’ and ‘rigid’ when its flexibility is 30% or less on lateral bending X-rays [2]. The treatment of these rigid curves frequently requires invasive interventions, such as osteotomy and operations with large resections of the posterior spine, which increase both the duration of the operation and the risk of complications, such as blood loss and neurological deficits. However, the benefits of such surgeries can be significant for aesthetics and overall health, helping to prevent further neurological deterioration [3].

Pediatric spinal deformities, including congenital scoliosis and kyphosis, can cause serious functional and aesthetic problems [1].

Amongst severe deformity etiologies, we may find idiopathic cases that exceed normal Cobb values, congenital deformities, deformities secondary to infectious diseases such as Pott’s disease, and neurological deformities [4]. These deformities are rigid and do not respond to conservative treatment or traditional arthrodesis techniques and posterior elements osteotomies. In these cases, more aggressive approaches are required, such as posterior vertebral column resection (PVCR), which offers effective correction of severe curves, allowing realignment of the vertebral structures even in complex situations [5,6]. PVCR consists of a complete resection of one or more vertebrae through a posterior approach, allowing intervention directly on the spine to correct angular deformities. This technique is particularly effective in patients with rigid deformities. In these patients, less invasive techniques are not adequate. It is important to emphasize that, although this procedure is necessary for these patients, it is technically complex and associated with high risks, such as significant blood loss and neurological complications [7,8,9].

There are several studies in the literature that have demonstrated the effectiveness of PVCR in the treatment of spinal deformities in different age groups, including pediatric, adolescent, and adult patients, but given the rarity of these cases, more reports are needed in order to emphasize the benefits and risks of such procedures. Our study aims to show the effectiveness and risks of this surgical treatment by detailing a case series performed at our institute.

## 2. Materials and Methods

This single-center case series evaluated prospectively collected data. Patients signed the informed consent for an observational study approved by the local Ethics Committee on 14 December 2016 (protocol number 0022814). Radiographic imaging was stored in an anonymized database.

### 2.1. Population

The study was conducted retrospectively on patients treated with vertebral VCR surgery for severe spinal deformities. All patients were assessed preoperatively using standard radiographs of the spine, magnetic resonance imaging (MRI), and computed tomography (CT) to determine the extent of the deformity and plan the surgery. Cobb’s angle was measured at all stages: pre-operative, postoperative, and during follow-ups. Patients selected for PVCR had a rigid deformity (flexibility < 10%) with a segmentary kyphosis of more than 50° Cobb, progression per year of more than 10/15° Cobb, and decompensation in the sagittal plane.

The study included a total of 7 pediatric patients undergoing surgery for the correction of severe spinal deformities treated at our center between January 2018 and December 2023. The population included 6 females and 1 male, with a mean age of 13.7 years (range: 11–17 years). All patients had complex spinal deformities, including scoliosis and kyphosis. The underlying pathologies included idiopathic kyphoscoliosis, neurofibromatosis type 1, Pott’s disease, and other congenital or syndromic anomalies. Intraoperative monitoring included the recording of motor evoked potentials to minimize the risk of neurological injury. Postoperative management included blood transfusions, antibiotic support, and early physiotherapy.

### 2.2. Surgical Technique for Posterior Vertebral Column Resection (PVCR)

The patient is placed prone on an operating table with careful padding of all salient pressure points. Neurophysiological monitoring, including somatosensory evoked potential (SSEP) and motor evoked potential (MEP), is applied prior to the procedure for monitoring spinal cord and nerve root function. A standard midline posterior approach is generally used and extended above and below the level of the deformity. We proceed with exposure of the posterior arches. At this point, free-hand pedicle screws are placed preferably at multiple levels because the closure of the osteotomy requires adequate vertebral purchase. Bilateral joint osteotomies are performed at adjacent levels to release the posterior column, improve spinal flexibility, and obtain fusion. Prior to the osteotomy, we ligated and cauterized segmentary arteries at the level of the osteotomy plus one above and below. All patients had preoperative angiography to identify the Adamkiewicz artery. Throughout the process, meticulous hemostasis was carried out. Double-lumen endobronchial tubes were part of the anesthesiologic setup. However, no patient had to have one lung excluded. To prevent lung damage, a thorough and delicate dissection was conducted across the vertebral body during the approach. Anterior vessels are then avoided by surrounding the vertebral body to be removed with large retractors and abundant sponges. Only then did we proceed with a laminectomy at the level of the planned resection. We prefer to perform decompression just before correction because, in case of evoked potential alterations, the spine can be promptly corrected by shortening, a maneuver that is universally considered protective for neural elements. This leads to the main part of the operation: the resection of the vertebral body, pedicles, and portions of the adjacent discs.

The posterior elements, transverse processes, and pedicles are removed using osteotomes, high-speed drills, and rongeur forceps (surgeon’s choice). Once freed, the anterior portion of the vertebral body is carefully excised in a fragmentary manner, protecting the spinal cord anteriorly and laterally.

**Figure 1:**
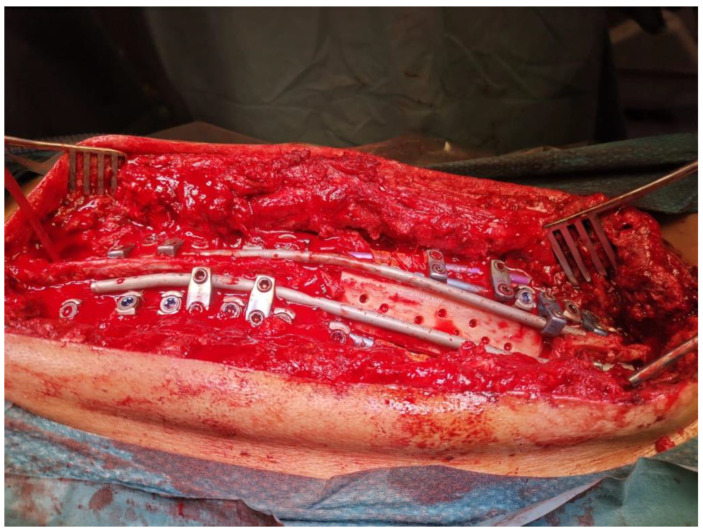
Posterior column reconstruction after PVCR with fresh frozen allograft.

Once the vertebral body is removed, segmental realignment of the spine is achieved by progressive compression at the resection site using the previously placed pedicle screws. Temporary rods can be used at this stage to maintain alignment and then further adjustments can be made as required. Once the correction is achieved, definitive rods are placed bilaterally to fix the correction by removing the temporary rods one at a time. In some cases, interbody grafts or structural supports may be inserted at the resection site to provide anterior column stability. Moreover, an autologous bone graft can be placed posteriorly to sit on the surface of the extremities of the spinous processes delimiting the posterior resection (Figure 1).

Hemostasis is crucial throughout the procedure, and blood scavenging during all surgery procedures included in our analysis was conducted with great care to minimize blood loss. The success of the posterior vertebral resection procedure depends on the meticulous execution of each surgical step, with particular attention to neurovascular structures and spinal stability during the procedure.

## 3. Results

### 3.1. General Population

Seven pediatric patients were included in the study, including six females and one male. The patients included in the study were of pediatric age, with a mean age of 12.5 years (range 10–16 years). The mean weight was 41.4 kg (range: 28–51 kg), and the mean height was 139.7 cm (range: 126–153 cm). The mean body mass index (BMI) was 21.1 (range: 13.3–26.1). Of these, one patient had scoliosis secondary to neurofibromatosis, one patient had kyphosis due to Pott’s disease (spinal tuberculosis), three patients had congenital kyphoscoliosis, one had post fibrocartilaginous mesenchymoma, and one patient had hereditary demyelinating polyneuropathy associated with kyphoscoliosis. The pre-operative Cobb angle average was 86.3° (range: 42°–140°). The postoperative Cobb angle was 22.4° (range: 12°–44°). The mean Cobb angle reduction was 62° compared to pre-operative values. The surgery resulted in a significant correction of the spinal deformity, with an average reduction of approximately 64% in the Cobb’s angle. The average duration of the interventions was 337.4 min (range: 190 min–480 min).

### 3.2. Complication

After surgery, three patients had an uneventful postoperative recovery, showing good clinical and radiographic outcomes during follow-up visits (Patients 1, 2, and 5). However, other patients experienced various complications that required careful management. Patient 3 developed a postoperative infection caused by E. coli, which was successfully treated with surgical debridement and antibiotic therapy with full recovery. Patient 4, on the other hand, experienced flaccid paraparesis, with loss of motor function predominantly on the right side immediately after the surgery. Following this, on postop day 6, she developed a febrile episode that required amine support. Despite the initial challenges, the patient received prompt rehabilitation treatment, leading to a gradual improvement in motor function. To continue her recovery, she was transferred to a rehabilitation unit for ongoing care. Patient 6 faced severe complications, including paraplegia and loss of sphincter control on postoperative day 6, attributed to an epidural hematoma at the site of PVCR at the thoracolumbar junction. Prior to the hematoma, she had a febrile episode. She had to undergo emergency surgery for hematoma evacuation, which resulted in gradual neurological recovery, although the patient remained catheterized due to bladder involvement. Finally, Patient 7 had a satisfactory postoperative recovery, with only a postoperative fever treated with antibiotic therapy. The postoperative complications, therefore, varied from infections (in three patients) to neurological issues (in two patients). Effective postoperative management proved crucial in addressing these complications and supporting patient recovery.

### 3.3. Patients

#### 3.3.1. Case 1

An 11-year-old female, weighing 51 kg and measuring 153 cm in height (BMI: 21.8), presented with a sudden onset of lumbar spine pain in June 2018.

##### Initial Diagnosis and Treatment

Initial imaging studies revealed vertebra plana at L1, with a hyperintense signal on STIR sequences and a hypointense signal on T1-weighted images. Additional findings included a hyperintense signal at the left calcaneus and a target-like lesion in the right tibia. A CT-guided biopsy of L1 was non-diagnostic. Based on the clinical presentation and imaging findings, a diagnosis of Langerhans cell histiocytosis (LCH) was suspected. The patient began treatment with vinblastine and prednisone in July 2018. Despite therapy, persistent radiographic uptake was observed, leading to an open biopsy of L1 at another facility. A histological analysis revealed an aneurysmal bone cyst (ABC) with focal eosinophilic inflammatory infiltrate, leading to the discontinuation of vinblastine and prednisone therapy. In February 2019, a repeat CT-guided needle biopsy of L1 at another institution provided a diagnosis of a “benign fibro-osseous lesion resembling fracture callus”. The patient continued conservative treatment with bracing. However, a biopsy of the right tibia in September 2019 showed “fibrino-hematic material encasing necrotic bone spicules, and remodeled trabecular and medullary bone”. Immunohistochemistry for Langerhans cells was negative. A new MRI in June 2020 showed resolution of the tibial lesion, but worsening kyphosis was noted despite conservative treatment.

##### Surgical Intervention

In February 2021, the patient was admitted to our spinal surgery department. The surgery lasted for a total of 3 h and 10 min. Posterior spinal stabilization was performed with pedicle screws inserted bilaterally at T11, T12, and L2. A vertebral column resection (VCR) was performed, and the gap was filled with homologous bone and biological cement. The removed material was sent to pathology, which required a second opinion for an accurate diagnosis. The final diagnosis was fibrocartilaginous mesenchymoma.

##### Postoperative Course

The patient had an uneventful postoperative recovery. Hemoglobin levels at admission were 10.7 g/dL, and at discharge, after two blood transfusions (one in the operating room and one in intensive care), hemoglobin was 11.5 g/dL. At the time of admission, the patient had a thoracolumbar kyphosis of 42°, with apex at the thoracolumbar junction. Postoperatively, the Cobb angle was reduced to 13°. At the most recent follow-up, 3 years and 6 months post-surgery, the patient reported no pain and showed good clinical and radiographic balance in both the coronal and sagittal planes.

#### 3.3.2. Case 2

##### Patient Overview

A 14-year-old male, weighing 28 kg and measuring 126 cm in height (BMI: 17.6), presented with right-convex congenital kyphoscoliosis, with predominant kyphotic deformity. Imaging revealed hemivertebrae from D10 to L2 and butterfly vertebrae at D9, L2, and L3, along with rib hypoplasia and a bifid rib branch. The patient had not undergone any prior surgical intervention or conservative treatment before surgical evaluation.

##### Surgical Intervention

The total duration of the surgery was 4 h and 7 min. A relatively short posterior spinal fusion was performed, with pedicle screws placed bilaterally at T7, T9, T10, L1, and L2, and unilaterally on the left side at T8. A bilateral costotransversectomy of T11 and T12 was performed, followed by a bilateral ligation of the T11 and T12 nerve roots. The lateral portion of the T11 vertebral body was exposed on both sides, and dissection was carried out to completely expose the anterior portion of the vertebral body. Using a high-speed burr and simple or ultrasound osteotomes, the T11 pedicle and vertebral body were resected. A temporary 5.5 mm rod was placed on the left side. A laminectomy of T11 and T12 was performed, followed by completion of the osteotomy, resulting in the segmental resection of T11 (vertebral column resection, PVCR). A 5.5 mm rod was then placed on the right side, the previous temporary rod was removed, and final corrective maneuvers both for kyphosis and scoliosis were performed on both final rods.

##### Postoperative Course

The postoperative course was uneventful, and the patient did not experience any complications. The preoperative hemoglobin was 13.6 g/dL, and following a single intraoperative transfusion, the hemoglobin level was 10.4 g/dL at discharge. Preoperatively, the patient had a thoracic kyphosis of 74° Cobb, which was reduced to 18° postoperatively. At the last follow-up, 1 year and 7 months after surgery, the patient reported no pain and demonstrated good coronal and sagittal balance both clinically and radiographically.

**Figure 2:**
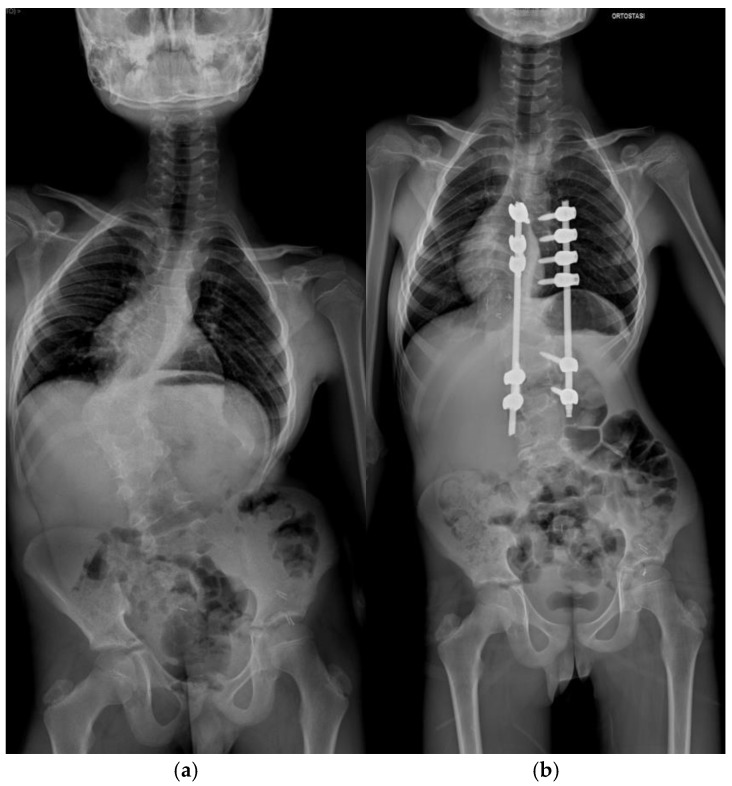
Antero-posterior radiograph of Patient 2. (**a**) Preoperative assessment. (**b**) Postoperative assessment.

**Figure 3:**
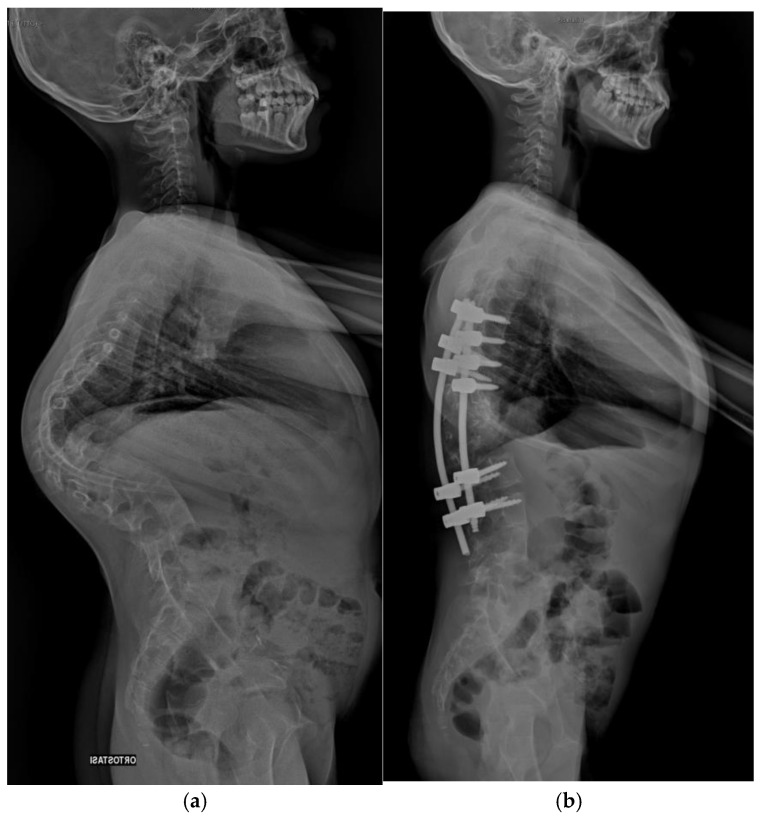
Lateral radiograph of Patient 2. (**a**) Preoperative assessment. (**b**) Postoperative assessment.

Pre-operative and post-operative radiographs documenting case 2 are reported in Figure 2 and Figure 3.

#### 3.3.3. Case 3

##### Patient Overview

A 14-year-old female, weighing 41 kg and measuring 141 cm in height (BMI: 20.6), was diagnosed with Pott’s disease (spinal tuberculosis) following a microbiologically confirmed vertebral biopsy in Ukraine in January 2017. She was placed on second-line anti-tuberculosis therapy, including pyrazinamide, ethambutol, cycloserine, prothionamide, levofloxacin, and capreomycin, which she continued for two years. Four years after the initial diagnosis, the patient presented to our clinic. Radiographic examinations revealed post-infectious intersomatic fusion from D12 to L5. Infectious disease consultation confirmed no active signs of infection, clearing the patient for surgical intervention.

##### Surgical Intervention

The surgery lasted 7 h and 40 min. Posterior spinal arthrodesis was performed, involving the placement of titanium pedicle screws bilaterally at L5, L4, L3, T11, T10, T9, and T8. A bilateral costotransversectomy was performed at T12, with an additional right-side costotransversectomy at T11. The transverse processes of L1 were resected bilaterally. Using a blunt dissection technique and ligation of the segmental arteries, the vertebral bodies from T12 to L2 were isolated, allowing for the placement of malleable retractors. Laminectomy and artrectomy from T11 to L2 were performed, exposing the dural sac. The T11 to L1 nerve roots were ligated and resected, and the spinal cord was protected using cottonoid sponges according to the “Chiripà” technique. Vertebral column resection (VCR) was performed at the apex of the deformity. Two rods were contoured and placed on the left side, connected with a domino connector. A progressive correction was achieved by a distraction of the anterior column and compression of the posterior column. A Pyramesh cage, filled with autologous bone graft, was inserted for anterior column support. Further deformity correction was accomplished with the compression of the posterior column, and the temporary rods were replaced with contoured titanium rods. The facet joints were decorticated, and posterior fusion was completed using an autologous bone graft. A “roof” structure was created using homologous cortical long bone to protect the neural elements, secured with a Jazz band.

##### Postoperative Course

During hospitalization, the patient experienced delayed wound healing with findings of E. coli, which was successfully treated with antibiotic therapy. Her preoperative hemoglobin level was 9.3 g/dL, and after two intraoperative blood transfusions, her hemoglobin at discharge was 11.7 g/dL. At the most recent follow-up, 3 years and 3 months post-surgery, the patient demonstrated good coronal and sagittal balance, with radiographic evidence of roof fusion. She reported no pain or neurological symptoms.

**Figure 4:**
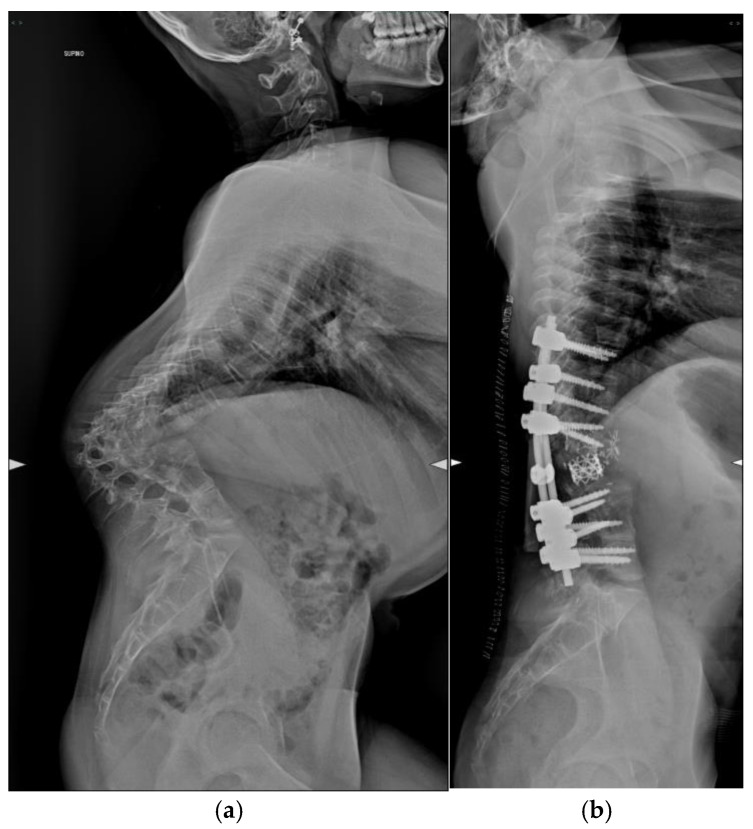
Lateral radiograph of Patient 3. (**a**) Preoperative assessment. (**b**) Postoperative assessment.

Pre-operative and post-operative radiographs documenting case 3 are reported in Figure 4.

#### 3.3.4. Case 4

##### Patient Overview

A 13-year-old female patient with neurofibromatosis type 1 (NF1) was evaluated in our outpatient clinic for severe thoracic scoliosis and kyphosis. The patient has a weight of 37 kg, a height of 138 cm, and a BMI of 19.4. Physical examination revealed a significant vertebral deformity, with hypoplasia of the T8 and T9 vertebrae, a painful left costal hump, and fatigue when walking. Following the clinical evaluation and anamnesis, a complete magnetic resonance imaging (MRI) and computed tomography (CT) scan of the spine was performed to rule out spinal cord malformations. The clinical findings and radiological images confirmed the need for surgical correction, for which a spinal fusion was indicated.

##### Surgical Intervention

The surgery lasted a total of 6 h and 20 min. A posterior spinal fusion from T2 to L1 was performed, involving the placement of multiple pedicle screws and two hooks at T2. Two rib segments were resected on the convex side and three rib segments on the concave side at the deformity apex. The vertebral body at the apex (T8) was isolated, followed by a wide laminectomy and vertebrectomy of T8. Anterior support was provided by a Pyramesh cage filled with autologous and synthetic bone grafts. Corrective maneuvers were performed, and definitive 5 mm titanium rods were placed. A rod-doubling technique was applied on the concave side using specialized connectors. Abundant washouts, decortication of the posterior fusion bed, and grafting with autologous and homologous bone were completed. A fibular allograft strut was positioned to bridge the laminectomy site on the concave side.

##### Intraoperative and Postoperative Course

During the vertebrectomy, a reduction in motor responses was observed, predominantly on the right side. Upon awakening, the patient exhibited flaccid paraparesis with more pronounced proximal weakness on the right side. Rehabilitation was initiated immediately in the intensive care unit. The patient developed a febrile episode, requiring amine support. Empirical antibiotic therapy with piperacillin/tazobactam was initiated. After intensive care, the patient returned to the general ward, where functional rehabilitation continued with progressive neurological improvement. By discharge, the patient could stand with assistance and support, and she was able to urinate independently. The surgical wound healed well and was closing by primary intention. The patient was transferred to a spinal rehabilitation unit for continued recovery. Preoperative radiographs revealed a 68° left thoracic scoliosis, which was reduced to 21° postoperatively. Hemoglobin levels dropped from 13.9 g/dL preoperatively to 10.6 g/dL postoperatively, requiring a single intraoperative blood transfusion. At the last follow-up (1 year and 3 months post-surgery), the patient was walking independently, including on her toes and heels, with excellent recovery after physiotherapy. A radiographic evaluation demonstrated a good correction in both coronal and sagittal planes.

#### 3.3.5. Case 5

##### Patient Overview

A 17-year-old female presented with a progressive thoracolumbar kyphotic deformity associated with multiple vertebral fusions. She had undergone conservative treatment during adolescence until her growth was complete. Rheumatological and genetic investigations were negative for specific conditions. Two years prior to admission, she began experiencing thoracic spine pain and difficulty with daily activities, including school-related tasks. More recently, she reported respiratory difficulties both while walking and lying supine.

##### Surgical Procedure

The surgery lasted 6 h and 7 min. A posterior instrumented spinal fusion from T4 to L4 was performed with the placement of pedicle screws from T4 to L4. Bilateral costotransversectomies at T10 and T11 were carried out at the apex of the deformity, with ligation of the nerve roots at these levels. The lateral and anterior walls of T10 and T11 were exposed, followed by a laminectomy at T10 and T11. Removal of the T11 vertebra was performed. The resected rib segments were placed as grafts between the vertebral bodies to bridge the vertebrectomy site. Titanium alloy rods were applied, and multiple corrective maneuvers were used to reduce the kyphosis, followed by compression closure of the osteotomy site. Rod doubling was performed with specialized connectors. An additional large amount of autologous bone graft was applied anteriorly, secured with a dual-mesh synthetic membrane and attached to rods and screws with non-absorbable sutures.

##### Postoperative Course

The postoperative course was uneventful. The patient was able to mobilize, achieving independent standing and walking. A thoracic surgery consultant reviewed chest X-rays and a CT scan without contrast, which showed moderate bilateral pleural effusion, but no indication for thoracentesis was given. Preoperative kyphosis measured 88° Cobb, which was reduced to 7° on postoperative radiographs. Preoperative hemoglobin was 12.7 g/dL, and at discharge, following two intraoperative blood transfusions, it was 11.6 g/dL. At the 1 year and 10 months follow-up, the patient was in excellent condition. Radiographs and CT scans showed solid graft fusion and a good balance in both coronal and sagittal planes.

**Figure 5:**
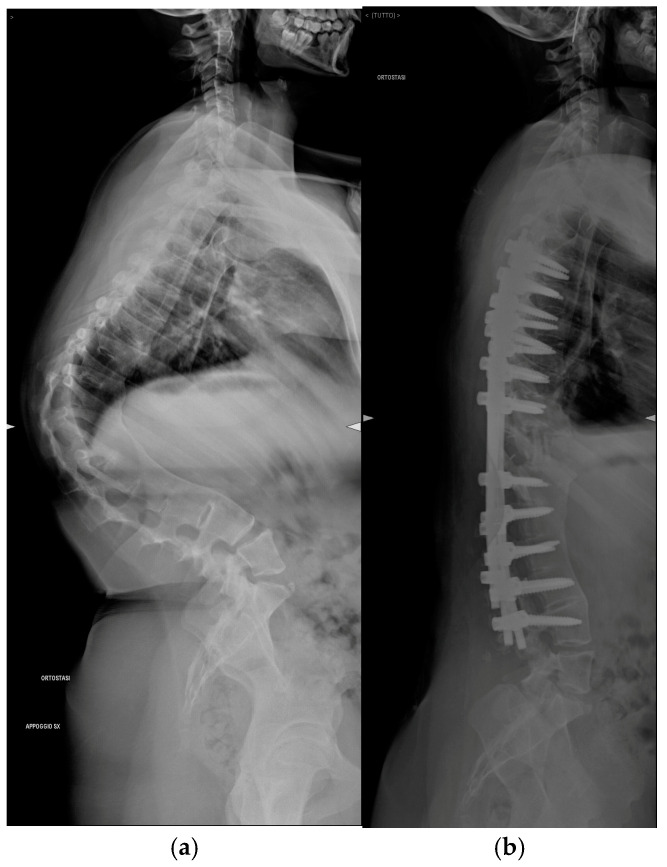
Lateral radiograph of Patient 5. (**a**) Preoperative assessment. (**b**) Postoperative assessment.

Pre-operative and post-operative radiographs documenting case 5 are reported in Figure 5.

#### 3.3.6. Case 6

##### Patient Overview

A 17-year-old female, weighing 49 kg and measuring 137 cm in height (BMI: 26.1), presented with severe congenital kyphoscoliosis measuring 140° Cobb. The patient had neurologic symptoms and ambulatory autonomy inferior to 50 m prior to surgery due to severe progressive deformity. Prior to surgery, the patient underwent Halo traction, gradually increasing the load up to 7 kg for 10 days. Halo treatment did achieve some correction, but it had to be suspended after 10 days because of worsening neurological lower-limb symptoms. Surgery was then performed, with a T11–T12 vertebrectomy and a posterior spinal fusion from T5 to L4.

##### Surgical Procedure

The total duration of the surgery was 5 h and 45 min. A posterior instrumented fusion was performed from T5 to L4, with pedicle screws placed from T5 to L4. Bilateral costotransversectomies at T11 and T12 were carried out, followed by the ligation of the nerve roots and segmental arteries at these levels. The vertebral bodies were isolated anteriorly, and a temporary stabilizing rod was placed. A laminectomy from T10 to L2 and vertebrectomies at T11 and T12 were performed. A Pyramesh cage filled with autologous bone graft was placed between the vertebral bodies, and progressive corrective maneuvers were employed to reduce both kyphosis and scoliosis. Final stabilization was achieved with definitive rods, including left-sided rod doubling via MRC connectors. A dual mesh was positioned to protect the anterior vascular structures. The system was secured, and posterior autologous bone grafting, supplemented by homologous bone (diaphyseal bone stick), was performed.

##### Postoperative Complications and Management

On postoperative day 5, the patient developed a fever of 39 °C. Blood and urine cultures were obtained, and based on an internal medicine consultation, Tazocin (piperacillin/tazobactam) was initiated. That night, the patient developed paraplegia and loss of sphincter control. Urgent CT and MRI of the spine revealed an epidural hematoma at the thoracolumbar junction (site of previous laminectomy), necessitating emergency surgery for hematoma evacuation. Cultures subsequently grew E. Coli, and the antibiotic regimen was adjusted to ceftriaxone, leading to the resolution of the infection. Postoperatively, the patient was closely monitored for neurological recovery. At discharge, she exhibited bilateral one-fifth strength in the iliopsoas muscles, three-fifths strength in the right tibialis anterior and flexor muscles, tactile hypoesthesia in the lower limbs, altered pain perception, and anal sphincter dysfunction. She remained catheterized due to bladder involvement, with a retained sensitivity in the S2–S4 region. Over time, she showed gradual motor recovery, with distal motor strength improving to three-fifths, while proximal strength remained weak at one-fifth. The patient began a rehabilitation program and was able to achieve standing. Despite early postoperative paraplegia, the patient demonstrated excellent neuromotor recovery and was discharged with a urinary catheter. She was referred to a specialized neuromotor rehabilitation facility. The preoperative hemoglobin was 11.8 g/dL, and the postoperative hemoglobin was 10.9 g/dL after two intraoperative transfusions. On the preoperative X-ray, the kyphosis was 140° Cobb, and the postoperative kyphosis was 8° Cobb on an X-ray. At the 2 years and 6 months follow-up, the patient is able to walk independently for short distances and uses crutches for longer walks. Her right leg shows greater strength compared to the left. Radiographic follow-up demonstrates maintained correction, with a stable kyphosis at 8° and a good spinal alignment. Unfortunately, the bladder function did not recover, and she has to perform urinary catheterisms, complicated by frequent urinary tract infections that still require medical attention and need surgical interventions in a urologic unit.

#### 3.3.7. Case 7

The patient is a 12-year-old girl, affected by severe and rapidly progressive scoliosis and kyphosis, with a weight of 35 kg, a height of 141 cm, and a BMI of 17.6. The presence of both severe scoliosis and kyphosis did not permit the application of growing rod instrumentations, so a single-stage posterior fusion was decided upon, despite the young age (the patient was pre-menarche). She has a genetic diagnosis of hereditary demyelinating polyneuropathy related to a heterozygous mutation of the SH3TC2 gene and suffers from chronic respiratory failure due to severe restrictive ventilatory deficit. This condition necessitated the initiation of a non-invasive ventilation (NIV) program to improve respiratory function.

##### Surgery

The surgery lasted 5 h and 50 min. VCR osteotomy of T8 and instrumented vertebral arthrodesis from vertebra T2 to L3 were conducted. During the operation, the vertebral column was exposed from vertebra T2 to L4, with facetectomies at all exposed levels and the insertion of pedicle screws and transverse hooks at T2. A costotransversectomy of T8 was performed, with removal of the costal segments of T8 and T9 and subsequent isolation of the vertebral body of T8. The removal of T8 was completed by protecting the cord with cotonoids (Chiripà technique). An anterior pyramesh was placed and filled with autologous bone graft, while 5.5 mm titanium rods were placed posteriorly to stabilize the spine. 

##### Postoperative Course

In the postoperative period, the patient developed a thermal rise, for which she was treated with piperacillin/tazobactam antibiotic therapy for 9 days, with good clinical and instrumental improvement. She started a rehabilitation program, which is to be continued at home according to instructions. Pre-operative X-ray showed severe thoracic hyperkyphosis of 100 degrees and scoliosis of 90 degrees, which, respectively, were reduced to 44 and 30 degrees. Pre-operatively, the patient had a hemoglobin of 15.1 g/dL. After a single intraoperative transfusion, the hemoglobin at discharge had dropped to 10.9 g/dL, a predictable drop given the complex surgery and intraoperative transfusion (Table 1).

## 4. Discussion

The management of pediatric spinal deformities, particularly scoliosis and kyphosis, represents a significant challenge for orthopedic surgeons. Recent publications highlight different surgical techniques and their results, contributing to a deeper understanding of their efficacy and safety. As already described, posterior vertebral resections (PVCR) have emerged as a key option for the treatment of severe spinal deformities. The radiographic data from our study show that severe, rigid, and poorly reducible kyphoscoliosis was indeed manageable with aggressive surgical treatment, yielding excellent radiographic and especially clinical results. Authors such as Ould-Slimane et al. (2022) and Hwang et al. (2019) point out how PVCR can correct rigid deformities, with a focus on comparing single- and multi-level resections [5,9].

Obviously, the choice of levels to be treated and the technique influence the clinical and radiological results. The type of surgical choice is in fact dependent upon the starting stiffness of the curve and the deformity.

Among the fundamental aspects to consider when talking about this type of surgery is the risk of complications. In fact, as we know, these are highly demolitive operations with an associated high risk of blood loss and intra- and postoperative neurological complications. The complications associated with PVCR are a crucial aspect to consider. As reported by Matsumura et al. [10], the management of congenital deformities often carries a higher risk of postoperative complications.

Surgical site infections are among the most common complications and can occur with varying degrees of severity. Obviously, the risk of infection increases with the complexity of the surgery. In our study, three patients presented with postoperative surgical infection. One of these presented a simple surgical wound dehiscence treated with antibiotic therapy. Two presented with a febrile flare-up that then responded to targeted antibiotic therapy. In one patient, almost concomitant with the febrile upsurge (treated with Tazocin) was the formation of a major hematoma, resulting in cauda syndrome. The patient was promptly taken to the operating theater and evacuated. The possibility of neurological damage is a major concern in this type of surgery. Direct or indirect (secondary to hematomas) injuries to spinal nerves or nerve roots can cause motor or sensory deficits. Such injuries have potentially disabling consequences. In two of our patients, we experienced alterations in nerve conduction. In the first case, during surgery, there was a reduction in potentials, with spontaneous recovery already during hospitalization. In the second case, the patient presented a secondary compression of the neurological structures following the formation of a hematoma, resulting in sensory–motor and sphincter alterations. She was quickly treated surgically and currently presents a slight asymmetry of strength between the two limbs. No spinal cord-related complications occurred in the study by Lenke et al. [11]. In two patients (8.5%), there was an alteration of the intraoperative neuromonitoring during correction, but the data returned to baseline.

In the study by Mourad Ould-Slimane et al. [5], 25% of the patients showed a significant decrease in the amplitude of the evoked potential. In two patients, there was a spontaneous resolution, in one case by monoplegia on awakening, with complete spontaneous resolution within 45 days (direct cage damage). In another patient, there was a permanent psoas deficit following resection of the L2 root. It is well-known that neurorestorative treatment before and after PVCR could be appropriate to improve rehabilitation protocols, as Guo X. et al. recently demonstrated [12].

In our study, no mechanical complications of the instrumentation were noted, although the follow-up available to us was short. In contrast, mechanical complications are widely described in the literature. In Lenke’s study [11], two patients underwent implant revisions, in one patient after 2 years following a deep infection and in the second case following implant prominence at 3 years follow-up.

Particularly in patients with severe deformities, there may be adverse effects on postoperative respiratory function. It is essential to assess lung function pre- and postoperatively to adequately manage any respiratory complications. In the study by Schroeder et al. [8], intraoperative complications included altered intraoperative potentials in two cases and a pleural injury.

In our study, no patients presented respiratory complications.

Blood loss during surgery is another significant complication. Surgeons must be prepared to manage potentially severe bleeding and consider the need for blood transfusions, especially in pediatric patients. Our patients all underwent at least one intraoperative transfusion. In the literature, we found the protocol of Ould-Slimane M. et al., who combined iron, folic acid, and erythropoietin to obtain a preoperative hematocrit > 45%, combined with intraoperative tranexamic acid and the use of an ultrasound scalpel to minimize blood loss and the need for transfusions, very interesting [5]. An experienced anesthesiologic team is fundamental in these operations. In the present study, perioperative blood losses were estimated by analyzing the change in hemoglobin levels pre- and postoperatively. For each patient, the difference between the hemoglobin value recorded before surgery (pre-operative Hb) and that recorded afterward (postoperative Hb) was calculated. The results show an average decrease in hemoglobin levels of 1.5 g/dL, suggesting a significant average blood loss during the operative period.

Studies such as that of Lenke et al. (2009) show that ISR for severe pediatric deformities offers lasting results, with significant improvements in deformity correction and postoperative quality of life [11]. A limitation of our study is the lack of quality-of-life assessment due to the retrospective nature of our analysis. Even though questionnaires were not administered, the patients have been evaluated in a comprehensive clinical assessment during outpatient follow-up visits. All patients recovered their baseline activities and their preoperative ambulatory status.

For this type of treatment, the choice of patients to be treated is crucial a priori. In fact, Song et al. (2022) compare outcomes between pediatric, adolescent, and adult groups, highlighting that differences in results may result not only from age but also from skeletal maturity and the nature of the deformity. This requires a multidisciplinary approach and careful surgical planning [6].

## 5. Conclusions

Posterior vertebral resection techniques offer significant promise in the correction of pediatric spinal deformities. Of course, before deciding on a PVCR, other less invasive surgical indications are evaluated, such as growing constructs in very young patients of temporary rod distraction combined with a two-stage surgery. Growing rod constructs nevertheless are limited to non-hyperkyphotic cases, and temporary rod distraction is mainly useful for long-range curves rather than short-range congenital deformities. PVCR is indicated especially in the case of highly progressive rigid deformities. Careful case-by-case evaluation is necessary, especially because of the associated high risk of morbidity. Ideally, surgery should be performed before the deformity begins to cause neurologic symptoms, as a PVCR upon a delicate spinal cord can be more demanding. Research continues to provide valuable data that can improve clinical outcomes and quality of life for patients. Although ours is a small case series, it can provide important data for such treatment. Long-term monitoring is needed to fully understand the impact of these procedures and to further refine surgical techniques.

## Figures and Tables

**Table 1:** Descriptive Table.

	AGE	SEX	BMI	ETIOLOGY	TIME OF SURGERY	COMPLICATION	HB PRE	HB POST	COBB PRE	COBB POST	FUP
1	11	F	21.8	fibrocartilaginous mesenchymoma	190 min	NO	10.7 g/dL	11.5 g/dL	42°	13°	42 M
2	14	M	17.6	congenital	250 min	NO	13.6 g/dL	10.4 g/dL	74°	18°	19 M
3	14	F	20.6	Pott	480 min	E. coli, which was successfully treated with antibiotic therapy	9.3 g/dL	10.7 g/dL	95°	15°	39 M
4	13	F	19,4	NF1	380 min	reduction in motor responses on the right side. Upon awakening, the patient exhibited flaccid paraparesis	13.9 g/dL	10.6 g/dL	68°	21°	15 M
5	17	F	21.0	congenital	367 min	NO	12.7 g/dL	11.6 g/dL	88°	7°	22 M
6	17	F	26.1	congenital	345 min	fever of 39 °C.--> paraplegia and loss of sphincter control, necessitating emergency surgery for hematoma evacuation	11.8 g/dL	10.9 g/dL	140°	8°	30 M
7	12	F	17.6	mutation of the SH3TC2 gene	350 min	thermal rise, for which she was treated with piperacillin/tazobactam antibiotic therapy	15.1 g/dL	10.9 g/dL	100°/90°	44°/30°	32 M

## Data Availability

Data supporting reported results can be requested from the corresponding author.
